# Seasonal Variation in ATP-Induced Retinal Damage in the Cone-Dominant 13-Lined Ground Squirrel

**DOI:** 10.1167/tvst.13.11.5

**Published:** 2024-11-07

**Authors:** Owen R. Bowie, Hannah M. Follett, Ching Tzu Yu, Chloe Guillaume, Phyllis M. Summerfelt, Nicole Manfredonia, Jenna Grieshop, Dana K. Merriman, Sergey Tarima, Joseph Carroll

**Affiliations:** 1 School of Medicine, Medical College of Wisconsin, Milwaukee, WI, USA; 2 Cell Biology, Neurobiology & Anatomy, Medical College of Wisconsin, Milwaukee, WI, USA; 3 Ophthalmology & Visual Sciences, Medical College of Wisconsin, Milwaukee, WI, USA; 4 Joint Department of Biomedical Engineering, Marquette University and Medical College of Wisconsin, Milwaukee, WI, USA; 5 Department of Biology, University of Wisconsin Oshkosh, Oshkosh, WI, USA; 6 Division of Biostatistics, Institute for Health and Equity, Medical College of Wisconsin, Milwaukee, WI, USA

**Keywords:** ground squirrel, optical coherence tomography, hibernation, cone photoreceptors, adaptive optics

## Abstract

**Purpose:**

To examine whether time of year (relative to hibernation emergence) influences the retinal degenerative effects of intravitreal injection of adenosine triphosphate (ATP) in the 13-lined ground squirrel (13-LGS).

**Methods:**

Eighteen (9 male, 9 female) 13-LGS in three experimental cohorts (early season, mid-season, late season) (*n* = 6 each) underwent baseline imaging using scanning light ophthalmoscopy (SLO) and optical coherence tomography (OCT). Animals then received a 10-µL intravitreal injection of 0.723 M ATP, followed by OCT and SLO imaging at 3, 10, and 21 days. Adaptive optics SLO (AOSLO) was performed in animals without retinal damage after the 21-day follow-up. Retinal thickness, choroidal thickness, and cone density measures were compared to values from wild-type controls (*n* = 12).

**Results:**

Five animals (four early season, one late season) showed retinal damage post-ATP injection (Fisher's exact test, *P* = 0.065). Animals with retinal damage displayed areas of disrupted retinal lamination on OCT. Any changes in OCT thickness were generally present on initial follow-up and resolved at later time points. Follow-up imaging with AOSLO on animals without retinal damage showed no significant differences in the cone mosaic topography from control eyes. Axial length was increased in mid-/late-season cohorts relative to early season (*P* = 0.0025 and *P* = 0.0007).

**Conclusions:**

In this pilot study, the 13-LGS appears more susceptible to ATP-induced retinal damage during the early season. Future studies adjusting dose based on ocular biometry may help elucidate the impact of time of year on chemical response.

**Translational Relevance:**

Consideration of ocular biometry in this and other animal models is merited when using intravitreal methods of chemical administration.

## Introduction

Damage or dysfunction within cone photoreceptors is the underlying mechanism of many irreversible human retinal disorders. The use of animal models that selectively induce cone degeneration therefore provides valuable models with which to examine pathological mechanisms underlying the development of human retinal diseases and explore potential avenues for their future treatment.^1^^–^^4^ Animal models, including rodents, rabbits, and felines, have been used in cone degeneration studies and offer a cost-effective and efficient means of producing rapid and often permanent retinal damage.^5^^–^^14^ Multiple methods involving intravitreal injections of chemicals, such as iodoacetic acid, tunicamycin, sodium nitroprusside, and adenosine triphosphate (ATP), have been investigated to induce selective cone photoreceptor degeneration. Through overactivation of ligand-gated purine receptors expressed in the eye (e.g., P2X7-R), ATP has been implicated in the mechanism underlying photoreceptor death when exposed to high extracellular concentrations.^15^ The use of intravitreal ATP has been demonstrated to produce selective photoreceptor damage while sparing other retinal structures.^9^ Previous studies using both feline and rodent models have demonstrated significant loss of retinal structure almost immediately following intravitreal administration of ATP, with additional signs of gross retinal degeneration and remodeling, such as neuronal and glial migration and proliferation, comparable to those observed in human retinal disease.^8^^,^^9^^,^^13^

The 13-lined ground squirrel (*Ictidomys tridecemlineatus*; 13-LGS) is an emerging animal model in translational vision research.^16^^–^^18^ These diurnal, cone-dominant (∼85% cones) animals have been used in studies of cone-based mammalian vision and provide particular benefit due to their amenability to high-resolution in vivo imaging modalities, including scanning light ophthalmoscopy (SLO), optical coherence tomography (OCT), and adaptive optics scanning light ophthalmoscopy (AOSLO).^19^^,^^20^ The 13-LGS is also an obligate seasonal hibernator that undergoes significant physiological changes and retinal remodeling during torpor, including migration of cone inner-segment mitochondria and disorganization of cone outer-segment discs, that rapidly recover upon emergence from hibernation.^16^^,^^17^^,^^20^^–^^24^ In addition, prior 13-LGS studies have examined retinal remodeling and recovery from damage induced through physical insults such as retinal detachment, as well as through intravitreal administration of cytotoxic chemicals such as ATP.^12^^,^^17^^,^^23^^,^^25^ While the feasibility of intravitreally injected ATP to create outer retinal layer–specific damage in the 13-LGS has been established previously, the damage induced varied significantly between animals receiving the same dose.^25^ Seasonal changes in 13-LGS physiology and retinal structure, as well as neuroprotection mechanisms associated with hibernating animals (e.g., tolerance of brain hypoxia/ischemia and metabolic stress), may provide a biological rationale for the variability seen in response to ATP.^26^ Here, we tested the hypothesis that the time of year (relative to emergence from hibernation) influences the degree of retinal damage in 13-LGS following intravitreal injection of ATP.

## Methods

### Animal Subjects

Eighteen adult (1 year old) 13-LGS (9 male, 9 female) were obtained from a breeding colony at the University of Wisconsin Oshkosh. Animals were randomly assigned to one of three cohorts (*n* = 6 animals each; 3 male, 3 female; 3 OD, 3 OS): early season (April, which is the typical time frame for emergence from hibernation), mid-season (June, which is the typical time frame for peak euthermic activity), or late season (August, which is the typical time frame for early hibernation immergence). Animal cohorts were balanced by sex due to differences in hibernation emergence time.^27^ An animal was reassigned to an alternate cohort if the initially assigned cohort contained a littermate. All animals had a coordinated hibernation exit date on April 1, 2021, with early-season animals receiving ATP injections 11 (*n* = 3) or 12 (*n* = 3) days later. One eye from each animal was randomly assigned to receive an ATP injection (*n* = 18 eyes; 9 OD, 9 OS) with the contralateral eye serving as an uninjected control. Control animals comprised uninjected control eyes of existing study animals and age-matched controls imaged throughout euthermia. Throughout the study, all 13-LGS were housed at the Medical College of Wisconsin according to previously described animal husbandry and dietary protocols.^27^ All procedures performed were approved by the Institutional Animal Care and Use Committee of the Medical College of Wisconsin (Protocol #AUA00005654) and in accordance with the ARVO Statement for the Use of Animals in Ophthalmic and Vision Research.

Prior to the procedures outlined below, animals were anesthetized with inhaled isoflurane (5% induction, 2%–4% maintenance) in oxygen (1 L/min) and positioned on a rodent alignment stage, using a protocol adapted from prior study.^25^ Artificial tears (Refresh Plus; Allergan, Irvine, CA, USA) were applied every 1 to 2 minutes throughout examination to maintain tear film and image quality. An ocular speculum was used to keep the eyelid open throughout each imaging session and during intravitreal injections. Respiratory rate and heart rate were monitored throughout anesthesia, and warming pads were used to maintain animal body temperature.

### Intravitreal ATP Injections

Intravitreal injection procedures were completed using an ophthalmic surgical microscope (Leica, Wetzlar, Germany) under bright illumination. After lubricating the eye with 2.5% Gonak hypromellose ophthalmic solution (Akorn, Lake Forest, IL, USA), a 6-mm circular cover slip (Fisher Scientific, Pittsburg, PA, USA) was placed over the cornea to improve visualization and ensure injection within the intravitreal space. Stabilization of the eye was maintained throughout the procedure manually by use of notched forceps. Intravitreal injections were performed using a sterile 10-mm, 30-gauge needle inserted approximately 1 mm posterior to the limbus. After insertion of the needle, each eye in the study received a 10-µL solution of sterile phosphate-buffered saline (0.15 M) containing 0.723 M adenosine triphosphate hydrate (Sigma Pharmaceuticals, VIC, Australia). Based on the results from a prior study, the dose of 10 µL 0.723 M ATP was selected to both minimize the amount of reflux of solution and produce widespread, robust degeneration selectively targeting the photoreceptor.^25^ All solutions were prepared the day of injection (within 1 hour of scheduled injection time) and stored protected from light and chilled at <4°C until time of use. Injection infusion was administered manually and slowly until complete volume was ejected from the syringe.

### Noninvasive Imaging

To prepare for each imaging session, one or both eyes were dilated and cyclopleged with one drop each of phenylephrine hydrochloride (2.5%) and tropicamide (1%). Additionally, tetracaine (0.05%) drops were given as topical eye surface anesthesia. SLO and OCT imaging was performed at baseline (prior to intravitreal ATP injection) as well as 3, 10, and 21 days postinjection, respectively. Contralateral control eyes were imaged at baseline with SLO and OCT. If observable retinal disruptions imaged with SLO were absent at 3, 10, and 21 days after contralateral ATP injection, OCT imaging was not performed. Baseline imaging sessions also included axial length measurements of injected and control eyes for each animal along the sagittal plane by ocular sonographic biometry (A/B-mode scan; Oscillator frequency: 10 MHz; Accutome A-Scan Plus Connect, Keeler, Malvern, PA, USA). Five measurements were taken, and the mean values were used for further statistical analysis.

### Scanning Laser Ophthalmoscopy

For subjective observation and monitoring of gross fundus changes, all eighteen 13-LGS retinas were imaged using a Spectralis SLO with a 55° lens (Heidelberg Engineering, Heidelberg, Germany). Multiple images were taken along the optic nerve head (ONH) with between 30% and 50% overlap spanning the entire length of the ONH (∼120°). Both near-infrared reflectance and short-wavelength autofluorescence modalities were used at all imaging locations.

### Optical Coherence Tomography

OCT imaging was performed using Bioptigen Envisu R2200 (imaging wavelength = 878.4 nm, bandwidth = 186.3 nm) or R2310 (imaging wavelength 860.4 nm, bandwidth = 111.7 nm) spectral domain OCT systems (Leica Microsystems, Wetzlar, Germany), using their rabbit lens. Prior to each imaging session, focus, reference arm position, and pupil centration were manually optimized. Both multiframe vertical line scans (100 B-scans) and rectangular volume scans (650 A-scan/B-scan, 300 scans) were taken sequentially along the optic nerve head (nasal to temporal or vice versa) until the entire nerve was imaged. Raw OCT images were used for analysis of retinal lamination thickness utilizing custom software (Duke Optical Coherence Tomography Retinal Analysis Program; DOCTRAP).^28^^,^^29^ OCT scans were uploaded into DOCTRAP, and layer boundaries were semiautomatically generated at the boundaries of the inner limiting membrane (ILM), outer nuclear layer boundary with the outer plexiform layer (ONL-OPL), retinal pigmented epithelium with choroid (RPE), and choroid–scleral interface (Ch) (Supplementary Fig. S1). Retinal layer thickness was assessed by matched eccentricity locations from 2.25° superior to the ONH and 16.50° inferior to the ONH within each scan: including total retinal thickness (TRT; ILM to Ch), inner retinal thickness (IRT; ILM to ONL-OPL), outer retinal thickness (ORT; ONL-OPL to RPE), and choroidal thickness (ChT; RPE to Ch). A custom MATLAB script (MathWorks, Natick, MA; https://github.com/AOIPLab/OCT_Thickness_Extractor/releases/tag/Bowie_et_al_2023) was used to calculate the average OCT thickness for each retinal layer to generate TRT, IRT, ORT, and ChT measures by retinal location. To generate thickness values by retinal location (degrees), thickness measurements were averaged across regions 0.75° wide and sampled from 2.25° superior to the ONH and 16.50° inferior to the ONH. Control animals utilized for OCT thickness comparisons were age-matched animals that were imaged previously throughout euthermia (*n* = 36 eyes) from a normative database of animals included in a previous study.^25^

### Adaptive Optics Scanning Light Ophthalmoscopy

Animals without evidence of damage on either SLO or OCT images were subject to AOSLO imaging within 1 month of completed follow-up imaging to determine if retinal damage or photoreceptor mosaic disruptions had occurred not discernible on prior imaging. AOSLO was not performed among the two undamaged animals in the early-season cohort due to them receiving retinal detachments as part of another study before AOSLO imaging could be completed.^30^ Animals with evidence of damage on either SLO or OCT imaging did not undergo additional AOSLO imaging since retinal damage had been established using less complex, lower-resolution methods. The AOSLO system used in this study was modified for a 4.5-mm pupil diameter and used a 97-actuator deformable mirror (ALPAO, Montbonnot-Saint-Martin, France), a Shack–Hartmann wavefront sensor (Rolera-XR; QImaging, Surrey, BC, Canada), and superluminescent diodes (Superlum, Carrigtwohill, County Cork, Ireland) for imaging (790.4 nm peak wavelength, 15.1 nm bandwidth) and wavefront sensing (845.8 nm peak wavelength, 26.9 nm bandwidth).^19^^,^^31^ Initial images of a Ronchi ruling at the start of each imaging session were obtained and processed to correct for the sinusoidal distortion imposed by the resonant scanner.^32^ Baseline SLO images were used as a reference for subsequent AOSLO imaging sessions. AOSLO videos were acquired initially at or above the ONH, followed by sequential overlapping images inferior, using retinal vessels as guides. Sequential videos were collected at multiple retinal locations with ∼50% overlap between adjacent videos.

Each raw AOSLO video was collected in confocal and non-confocal modalities and processed to generate high signal-to-noise ratio images for subsequent analysis. Extracted images from each video location were created, averaging 50 to 75 frames, using a custom registering and processing software, as described previously, which were semi-automatically aligned to create montages of the cone mosaic.^33^^–^^35^ Manual adjustments were performed in Photoshop (Adobe, San Jose, CA, USA) to fill gaps and correct alignment of overlapping images as needed to create the final montage.

Regions of interest (ROIs) were extracted from each montage using custom software (Mosaic Analytics, Translational Imaging Innovations, Inc., Hickory, NC, USA). Each ROI was 1° × 1° and sampled at 0.75° intervals from 2.25° superior to the ONH to 16.5° inferior to the ONH, for a total of up to 19 ROIs per animal. Cone photoreceptors within each ROI image were then semi-automatically counted in the non-confocal split-detection modality by a single observer (JC) using custom software (Mosaic Analytics) to obtain cone density estimates as previously described.^36^ Cone mosaic geometry in mid- and late-season animals was assessed using bound-cell density (cells/degree^2^) and voronoi cell area regularity (VCAR).^36^ Only cones within their corresponding voronoi cell fully contained within the ROI (i.e., “bound”) were considered for metric calculations.^36^ These measurements were compared to values from a data set of age-matched control animals by eccentricity (*n* = 12 eyes). VCAR was assessed for all animals at 6.25° inferior to the ONH (representing the location nearest the visual streak for which we had density values in all animals).

### Statistical Analysis

All statistical analyses were performed using GraphPad Prism 9 (GraphPad Software, San Diego, CA, USA) and RStudio (RStudio, PBC, Boston, MA, USA) with the vegan statistical package. Shapiro–Wilk tests were used to assess normality of each data set prior to statistical testing. To check for possible type I errors, we also inspected Q-Q plots for each data set, which confirmed nonnormality in some cases (Supplementary Fig. S2). Fisher's exact test was used to evaluate differences in the frequency of damaged animal means between cohorts, accounting for small sample sizes. Nonparametric tests, including the Friedman test and permutational multivariate analysis of variance (PERMANOVA), were used to analyze retinal thickness. Post hoc Wilcoxon tests were applied to further explore significant differences detected across time points and between groups. One-way analysis of variance (ANOVA) was used to assess differences in cone density root mean square error (RMSE) and cone mosaic VCAR among mid- and late-season animals compared to uninjected controls. A one-way ANOVA with a post hoc Tukey's multiple comparisons test was also performed to compare axial length measurements between cohorts.

## Results

### Retinal Changes Observed on SLO and OCT Imaging Between Seasonal Cohorts after Intravitreal Injection

**Figure 1.**
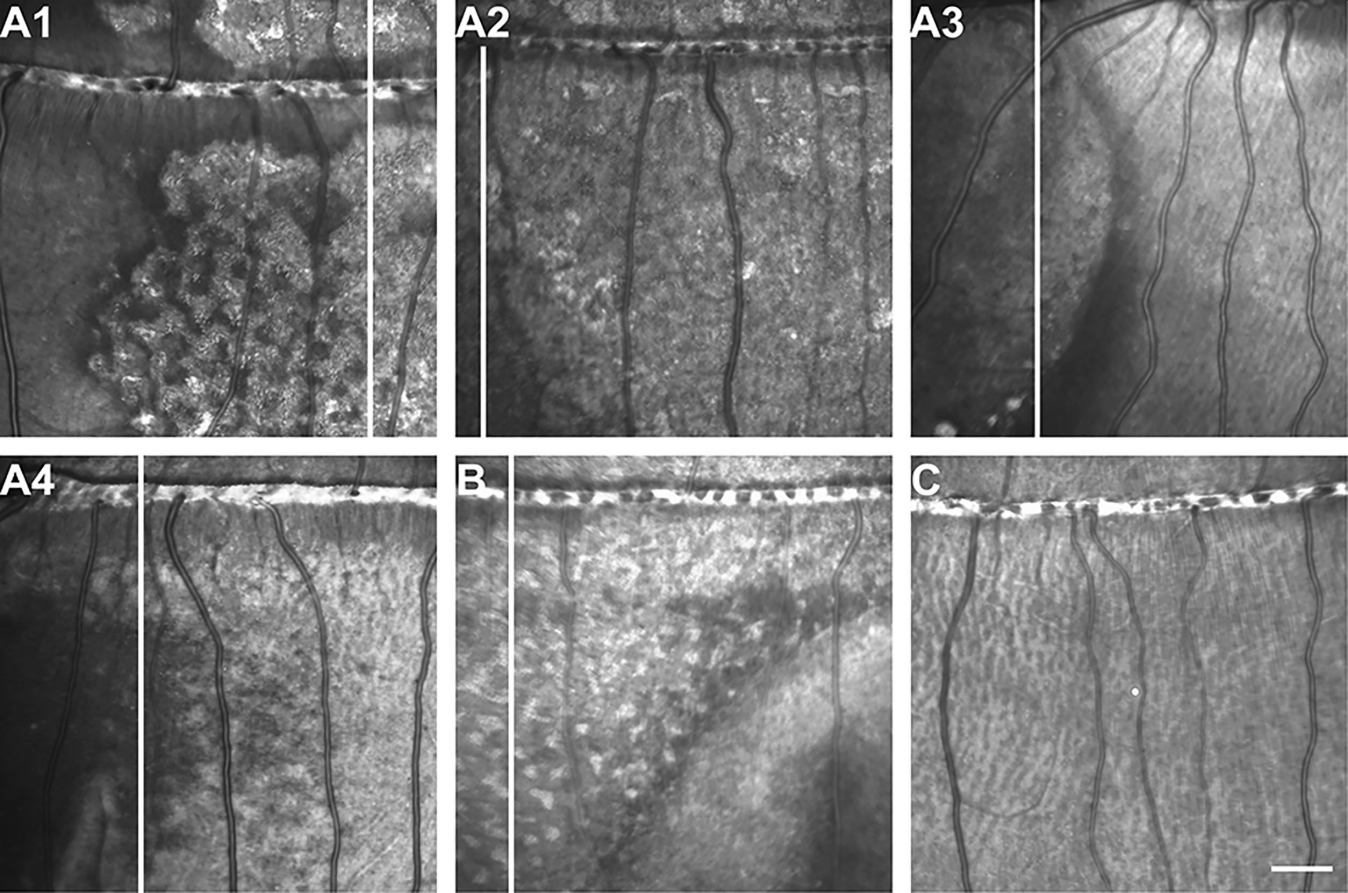
Differences in retinal damage observed following ATP intravitreal injection compared to preinjection animals. Shown are scanning laser ophthalmoscopy near-infrared images of the four damaged early-season animals (**A1–A4**), the damaged late-season animal (**B**), and preinjection baseline control images (**C**). Images from A1, A2, A4, and B were taken 3 days postinjection, while A3 was taken 10 days postinjection (damage was not observed on 3-day imaging). *White bars* represent location of OCT scans in Figure 2. *Scale bar*: 5° (lateral).

**Figure 2.**
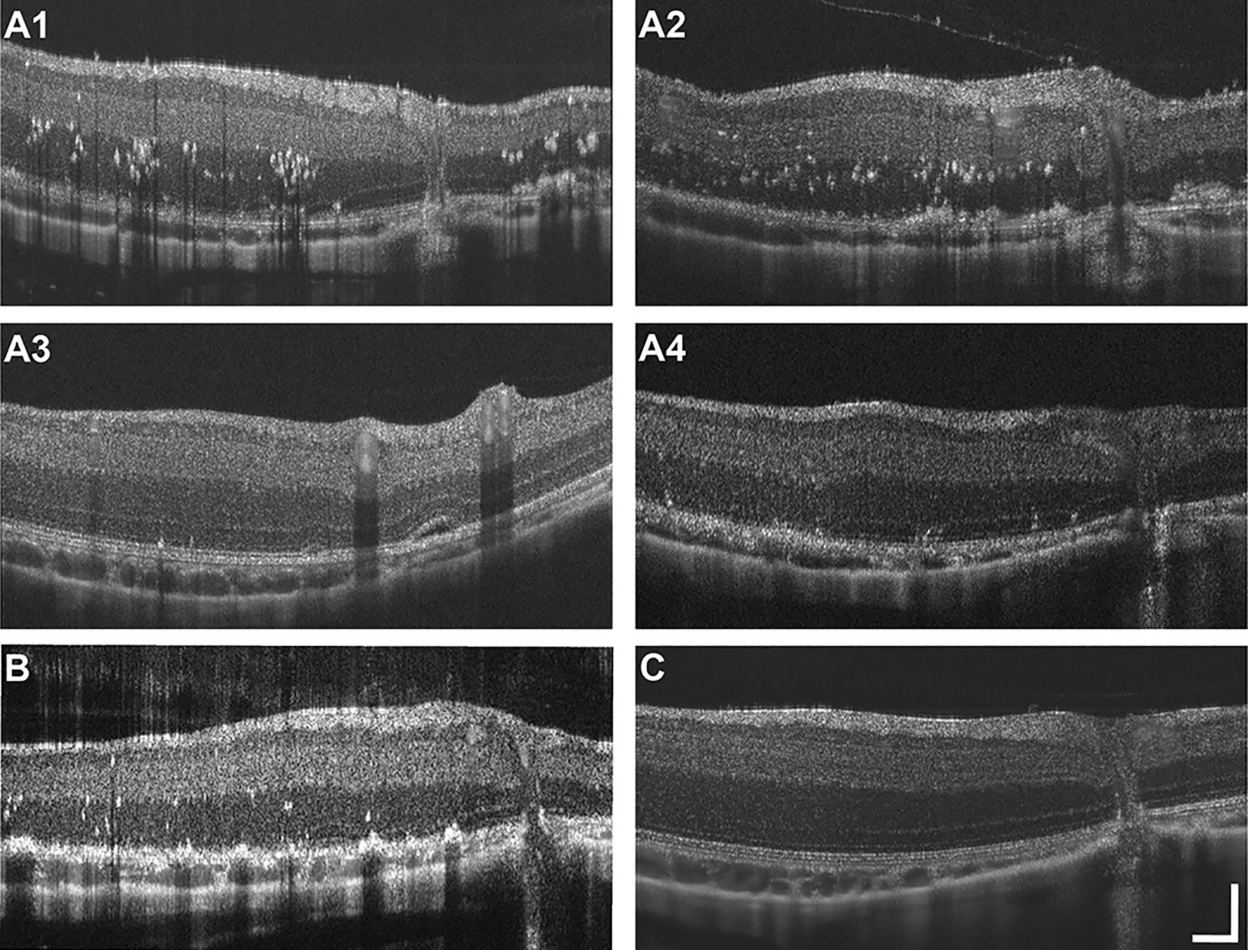
Differences in retinal damage observed following ATP intravitreal injection compared to preinjection animals. Hyperreflective puncta and retinal lamination disruptions were observed after intravitreal ATP injection at the 21-day follow-up, most notably in the outer retina and choroid, in early-season (**A1–A4**) and late-season (**B**) animals. These features were not observed in any of the preinjection control animal images (**C**). Retinal locations correspond to *white bars* in Figure 1. *Scale bar*: 1° (lateral) and 50 µm (axial).

None of the study animals displayed any abnormal phenotype or retinal disruptions at baseline prior to ATP injection, as assessed with SLO (Fig. 1C) and OCT imaging (Fig. 2C). In total, 5 of 18 animals demonstrated postinjection damage across all cohorts, including 4 in the early-season cohort and 1 in the late-season cohort. There was not a significant effect of time of season on frequency of retinal damage (*P* = 0.065, Fisher's exact test). One animal (DM_204205) in the early-season cohort did not display damage observable via SLO or OCT until 10 days postinjection, and damage was localized to the temporal retina (Fig. 1A3). Damage observed in another early-season animal, DM_205402 (Fig. 1A4), was also more strongly observed in the temporal retina, but it encompassed a larger relative area compared to damaged observed in DM_204205 (Fig. 1A3). Remaining early-season animals (DM_203104, DM_203503, and DM_204304) demonstrated damage on SLO more globally throughout the retina (Fig. 1A1, A2, B). In DM_203503, hyper- and hyporeflectivity changes were noted globally both superior and inferior to the ONH (Fig. 1A2). In DM_203104, hyporeflective changes were noted across the ONH, most notably in the temporal retina, with various patches of hyper- and hyporeflectivity globally throughout the retina (Fig. 1A1). These changes were like those seen in the one late-season animal, DM_204304, except that the latter's most notable hyporeflectivity was at the nasal retina along inferior vessels (Fig. 1B). Damage patterns on SLO did not change throughout the 21-day postinjection follow-up imaging.

When compared to baseline OCT among early- and late-season animals, hyperreflective puncta and retinal lamination disruptions were observed, most notably in the outer retina and choroid at the 21-day follow-up (Fig. 2). However, these changes were not observed in any of the mid-season animals who otherwise remained indistinguishable from baseline throughout follow-up imaging. Similarly, other undamaged animals in the early-season (*n* = 2) and late-season (*n* = 5) cohorts did not show appreciable differences compared to baseline imaging on OCT. Animals with SLO reflectivity changes observed more globally throughout the retina (Fig. 1A1, A2, B) also had the most prominent hyperreflective puncta noted in the outer retina on OCT, particularly by 21 days postinjection (Fig. 2A1, A2, B).

### Transient Increased Retinal and Choroidal Thickness in Early Follow-Up Relative to Uninjected Controls

**Figure 3.**
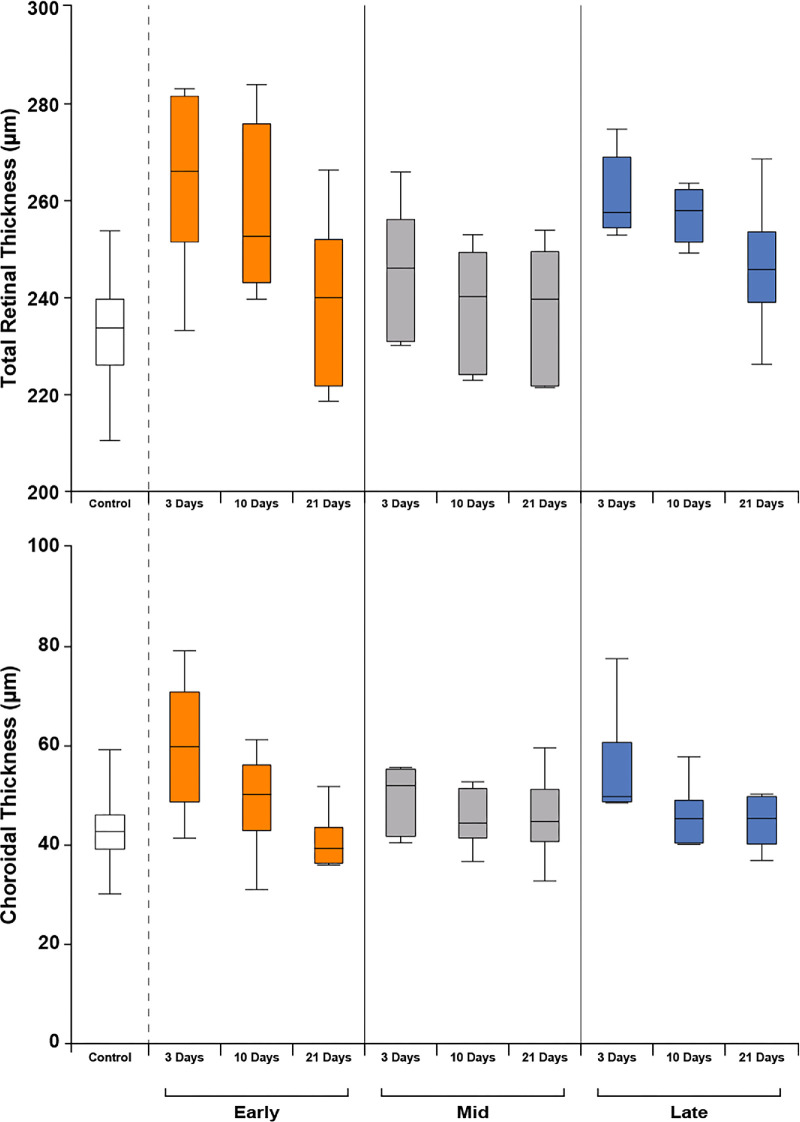
Total retinal thickness and choroidal thickness varied over time within and between seasonal cohorts. Average TRT varied significantly across follow-up time points within all three seasonal cohorts (early: *P* = 0.0046; mid: *P* = 0.0018; late: *P* = 0.0056). While TRT variance between early- and mid-season groups differed significantly (*P* = 0.048) and variance between early- and late-season groups trended toward significance (*P* = 0.055), no significant difference in TRT variance was observed between mid- and late-season groups (*P* = 0.551). Average choroidal thickness differed significantly across follow-up time points within the early-season group (*P* = 0.008) but not mid-season (*P* = 0.516) or late-season (*P* = 0.062) groups.

Differences in average TRT and ChT measures were observed between seasonal cohorts and across follow-up (Fig. 3). When compared to uninjected control average TRT (233.1 µm), both early-season (264.5 µm) and late-season (260.8 µm) average TRT was greater at the 3-day follow-up. These differences remained at the 10-day follow-up in both cohorts. All three cohorts demonstrated the trend of decreasing TRT throughout follow-up, most prominently in early- and late-season cohorts (Fig. 3). Analysis with Friedman tests confirmed significant differences in average TRT throughout follow-up time points relative to controls for all three seasonal cohorts (early: χ² = 13, *df* = 3, *P* = 0.0046; mid: χ² = 15, *df* = 3, *P* = 0.0018; late: χ² = 12.6, *df* = 3, *P* = 0.0056). Additional characterization revealed that these changes in TRT were due primarily to increases in inner retinal thickness (Supplementary Fig. S3). The average ChT among early-season animals at the 3-day follow-up was reduced from uninjected controls. However, at 10 and 21 days, the average ChT for early-season animals was similar to uninjected controls. The ChT in both the mid- and late-season animals was similar to uninjected controls throughout follow-up (Fig. 3). Friedman tests showed average ChT differed significantly across follow-up time points in the early-season cohorts (χ² = 11.8, *df* = 3, *P* = 0.0081) but did not differ significantly in mid-season (χ² = 2.28, *df* = 3, *P* = 0.5164) or late-season (χ² = 7.32, *df* = 3, *P* = 0.0624) cohorts.

### Seasonal Variation in Retinal Thickness

To assess the variance in total retinal thickness between seasonal cohorts, pairwise PERMANOVA analyses were performed. The comparison between the early- and mid-season groups revealed a marginally significant difference (*P* = 0.0476). Comparisons between early- and late-season cohorts showed a trend toward significance (*P* = 0.0554), whereas comparisons between mid- and late-season groups were not significantly different (*P* = 0.5506). Post hoc Wilcoxon–Mann–Whitney tests were performed to compare retinal thickness between the early-season group and the mid/late-season groups at various time points. Significant differences were observed between early- and mid/late-season groups at day 3 (W = 24, *P* = 0.0159) and day 10 (W = 25, *P* = 0.0079). By day 21, no significant difference was found between the early- and mid/late-season groups (W = 19, *P* = 0.2222).

### Individual Variability in Altered Retinal Lamina Thickness in Early- and Late-Season Animals Compared to Uninjected Controls

**Figure 4.**
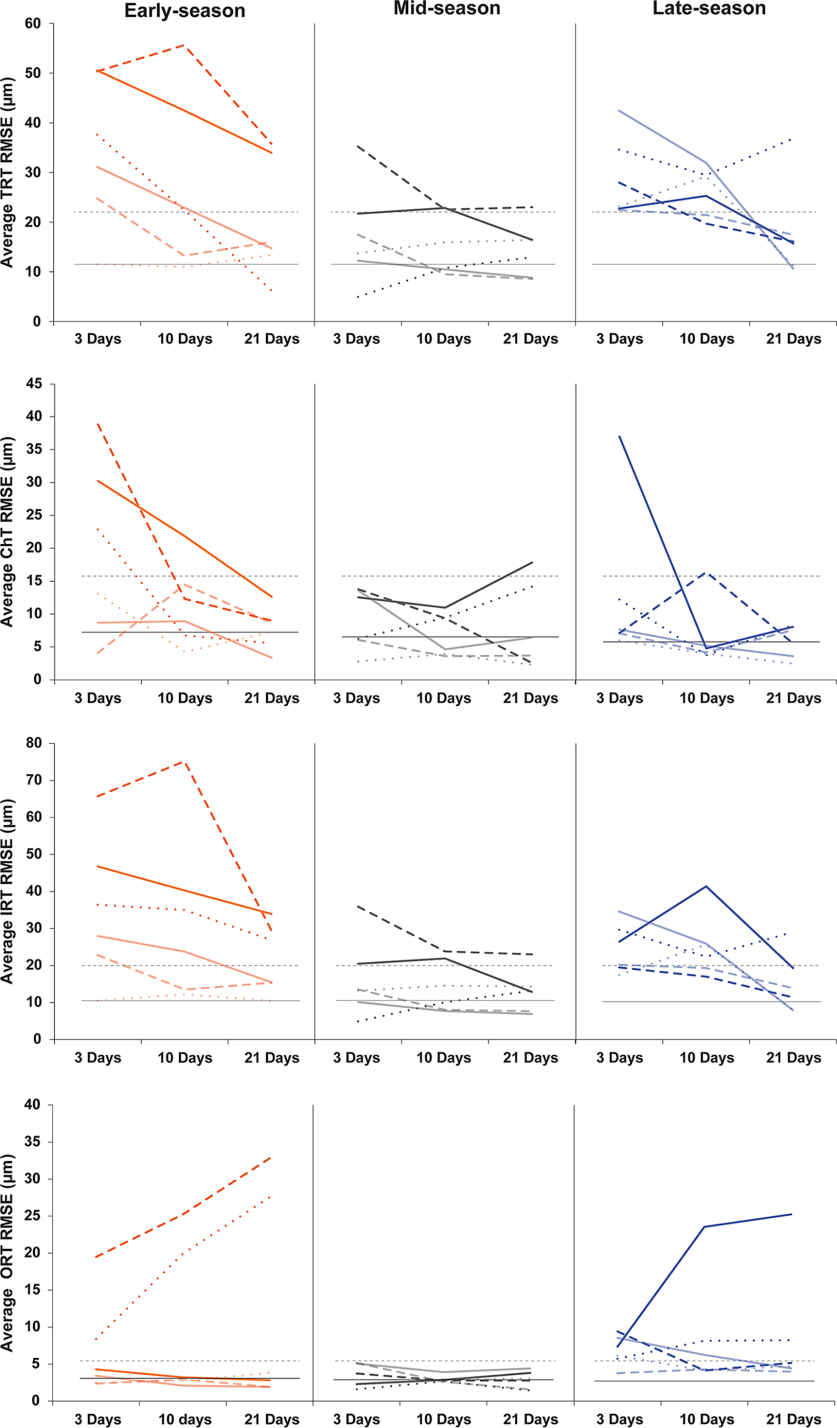
Individual animals showed greater variability in retinal and choroidal thickness from the average uninjected control. Each line is from a different animal. The *solid horizontal line* represents the average RMSE of the uninjected control animals (*n* = 36), while the *dashed line* represents the upper 95% confidence interval above the mean (mean + 1.96 * SD) of uninjected control animal RMSE. Mid-season animals showed RMSE values within those of uninjected controls in all retinal layers throughout postinjection follow-up. In general, early-season animals had increased RMSE at 3 days postinjection in all retinal layers, returning to within uninjected control values by 21 days. The exception was ORT, where two early-season animals showed increasing RMSE from 3 to 21 days postinjection (these same animals showed persistent increased TRT and IRT at 21 days postinjection). Late-season animals showed similar trends to early-season, with less variability. Like early-season animals, ORT for one late-season animal showed continued increased RMSE at 21 days postinjection.

We next assessed individual animals, comparing the RMSE for each layer in each animal against the average uninjected control value for that layer. Mid-season animals generally showed RMSE values within those of uninjected control animals (Fig. 4), with only seven measurements showing elevated RMSE values across TRT, ChT, and IRT (no ORT RMSE values fell outside normative values). In contrast, early-season animals showed highly variable thickness changes compared to uninjected control animals throughout all retinal layers (Fig. 4). Generally, early-season animals had increased RMSE at 3 days postinjection, which returned closer to uninjected control values by 21 days postinjection. The exception was ORT, where two early-season animals showed a continued increase in RMSE over time (these same two animals showed persistent increased RMSE for TRT and IRT at 21 days postinjection as well). Late-season animals showed similar trends as early-season animals but with less variability. All late-season animals had increased TRT RMSE at 3 days postinjection, with all but one animal returning to within normal values by 21 days postinjection (Fig. 4). The same animal showed persistent increased RMSE for ORT at 21 days postinjection. Calculated RMSE values by animals within each cohort are available in Supplementary Table S1.

### Normal Cone Mosaic Topography in Undamaged Mid- and Late-Season Animals

**Figure 5.**
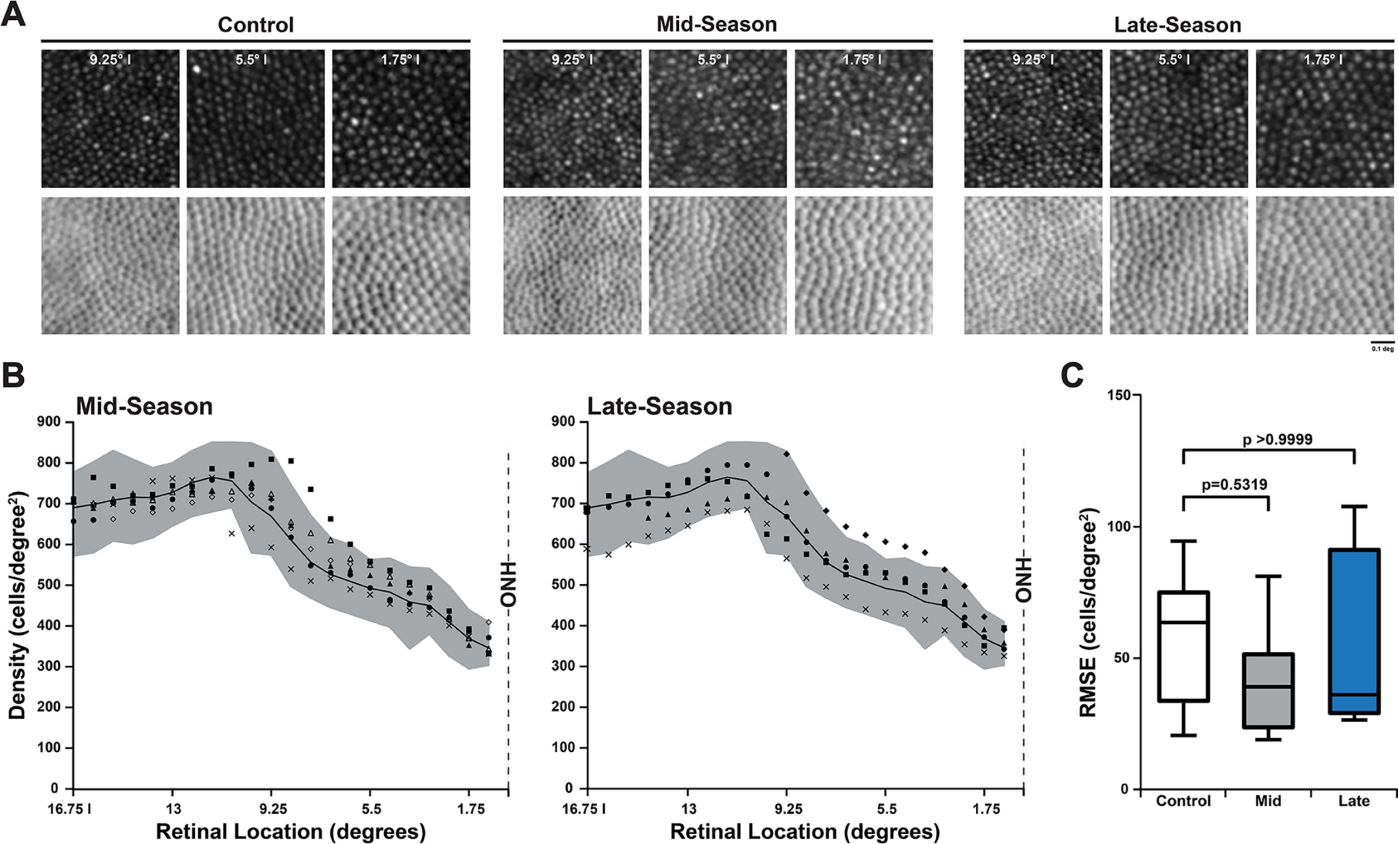
Animals without observed retinal damage after 21-day follow-up had normal cone photoreceptor topography. (**A**) AOSLO images at three matched eccentricities inferior (I) to the ONH at 9.25°, 5.5°, and 1.75° I showed a contiguous photoreceptor mosaic with normally reflecting cones among control (*left*) compared to mid-season (*middle*) and late-season (*right*) animals. (**B**) Bound-cell density measurements (cells/degrees^2^) from 16.75° I to the ONH among undamaged animals in mid-season (*left*) and late-season (*right*) cohorts; 96.97% of mid-season and 88.66% of late-season locations sampled were within the normative range (mean ± min/max; *gray area*) of control animals (*n* = 12 eyes). (**C**) Box plots of RMSE of mid- and late-season animals compared to controls show no significant difference. Similarly, RMSE of mid- and late-season animals was not significantly different (*P* = 0.53) by one-way ANOVA.

All AOSLO ROIs among mid-season (132 ROIs; *n* = 6) and late-season animals (97 ROIs; *n* = 5) showed a normal contiguous mosaic of normally reflecting cones after the 21-day follow-up (Fig. 5A). Bound-cell density measures for both cohorts were similar to controls at all locations (Fig. 5B), with 96.97% of mid-season and 88.66% of late-season ROIs having density values within control values. Interestingly, all but one of the observed density outliers were higher than normal, which would not be anticipated in the setting of photoreceptor damage. Cone density RMSE values from the mid- and late-season cohorts passed Shapiro–Wilk normality testing (mid: W = 0.85, *P* = 0.17; late: W = 0.85, *P* = 0.18), and no significant difference from control RMSE values was detected (one-way ANOVA; *F*(2, 20) = 0.68, *P* = 0.53) (Fig. 5C). VCAR values from each cohort passed Shapiro–Wilk normality testing (mid: W = 0.86, *P* = 0.18; late: W = 0.97, *P* = 0.86). The average VCAR at 6.25° inferior to the ONH was 11.27 for mid-season and 10.04 for late-season animals, which were not significantly different from control VCAR of 10.56 (one-way ANOVA; *F*(2, 20) = 0.77, *P* = 0.48).

### Axial Length Differences Observed Between Seasonal Cohorts

**Figure 6.**
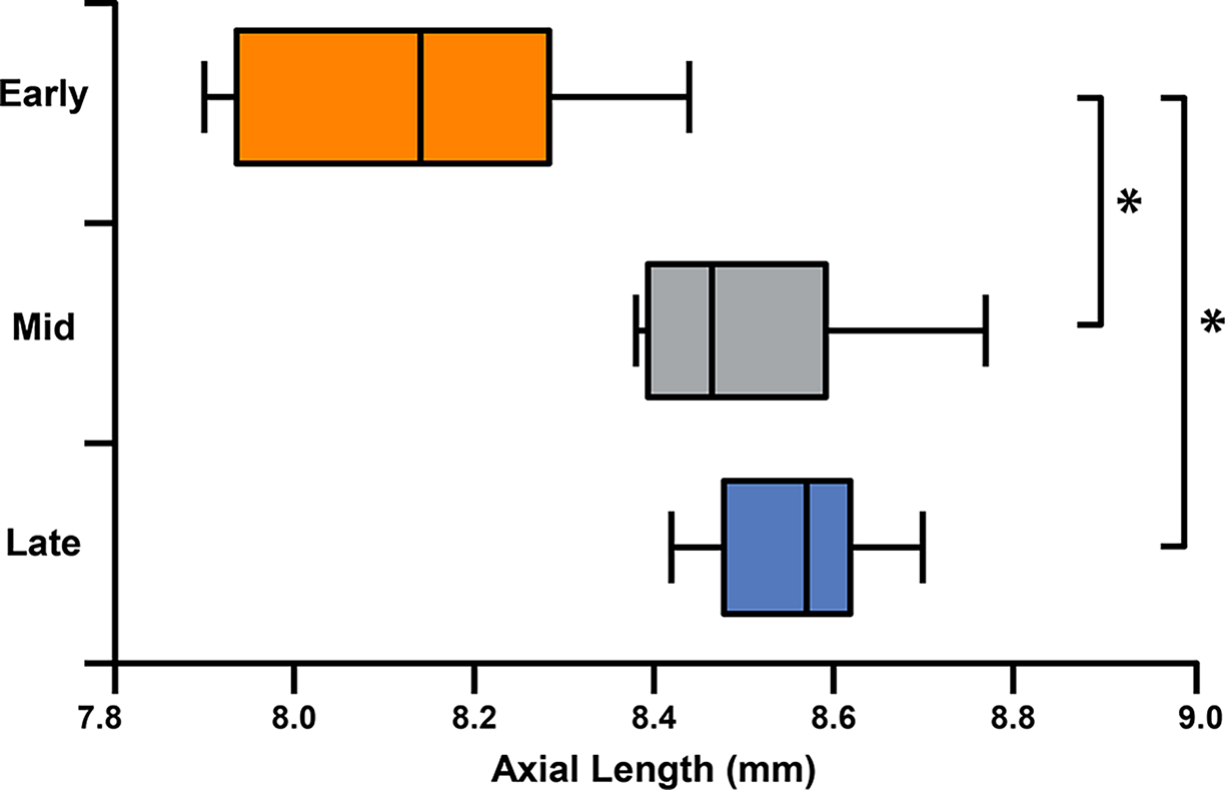
Axial length varied between cohorts. Average axial length observed among mid- and late-season animals was significantly different from early-season animals. Box plots of axial length differences between early- and mid/late-season cohorts were significant by one-way ANOVA (*P* = 0.0025 and *P* = 0.0007, respectively). Axial lengths between mid- and late-season cohorts were not significantly different (*P* = 0.8055).

Axial length measurements were obtained once (during baseline imaging sessions) and compared between seasonal cohorts (Fig. 6). Measurements from each cohort passed normality testing (early: W = 0.95, *P* = 0.75; mid: W = 0.84, *P* = 0.12; late: W = 0.98, *P* = 0.96). Early-season animals had a shorter axial length on average (8.13 ± 0.20 mm) compared to mid-season (8.50 ± 0.15 mm) and late-season (8.56 ± 0.10 mm) animals. A significant difference in axial length measurements between cohorts was detected by one-way ANOVA (*F*(2, 15) = 13.21, *P* = 0.0005), and a post hoc Tukey's multiple comparisons test showed the average axial length for the mid- and late-season cohorts was significantly greater than the early-season cohort (*P* = 0.0025 and *P* = 0.0007, respectively), whereas no significant difference was found between mid- and late-season cohorts (*P* = 0.8055) (Fig. 6).

## Discussion

Although four of the five animals that demonstrated postinjection damage were within the early-season cohort, there was not a statistically significant effect of time of euthermic season on the frequency of retinal damage. However, despite lacking a statistically significant difference (Fisher's *t*-test *P* = 0.065), this represents a potential effect worth evaluating further. Additionally, findings of marginal significance and trends of significance in the average TRT variance between early- and mid-season cohorts (*P* = 0.0476) and early- and late-season cohorts (*P* = 0.0554) support further investigation into the influence of season on the variation in ATP-induced retinal degeneration in 13-LGS. However, components of the study, including small sample sizes and the variability inherent to ATP injection, make it difficult to justify a conclusive statement on the presence or absence of postinjection damage. Regardless, there are factors that may contribute to the variability in response to ATP. For example, significant remodeling of the 13-LGS retina in early season, in response to hibernation emergence, may explain some of this variation observed between cohorts.^17^^,^^21^^,^^22^^,^^24^ It is possible that some animals may have been in the process of undergoing retinal remodeling while receiving ATP injections within the early-season cohort. Intravitreal injections given within this period may be more effective at producing retinal damage. However, when hibernation is terminated, 13-LGS retina cells recover within hours to days, with rapidly reversible plasticity.^17^^,^^22^^,^^24^

Alternatively, retinal morphology changes that occur specifically in the late season of 13-LGS to prepare for hibernation could be protective from retinal damage postinjection. Hibernating mammals develop protective mechanisms to prevent cell death during hibernation, including limiting excitotoxicity in response to cerebral ischemia.^26^ Retinal remodeling in the 13-LGS in preparation for hibernation could decrease susceptibility to oxidative damage.^17^ Notably, compared to prior studies in felines and rats (which do not hibernate), the initial pilot study using intravitreal ATP in 13-LGS required higher concentrations of ATP to induce retinal damage.^8^^,^^13^^,^^25^ Likewise, the result of few animals responding with even a moderate degree of retinal degeneration in this study was not expected. While the frequency of damage occurrence across cohorts suggests seasonal changes may drive some variability, the range of damage observed within the early season implies additional factors have a significant effect on the extent of degeneration induced. This may include inadequate dosing as a result of differing volumes of reflux, deterioration of the ATP solution, and differences in the effective dose at the retina due to the volume of vitreous the injected solution diffuses through. Consequential studies that determine a more accurate dose–response within the 13-LGS to intravitreal ATP may provide a more reliable response, regardless of time of season.

Early-season animals had significantly shorter average axial lengths at baseline compared to mid- and late-season animals. Seasonal physiological differences, such as significant dehydration and nutritional status following emergence from hibernation, may account for these observations.^17^^,^^20^ Axial length has been used as a measure in other studies as an analogous method to approximately measure vitreous volume.^2^ Relatively small differences in axial length equate to large differences in vitreous volume. For example, a 0.5-mm difference between 13-LGS eyes would equate to an approximately 20% difference in vitreous volume. The difference in measured axial length between animals ranged from 0.01 to 0.87 mm. As such, the absolute dose of chemical experienced by the retina could be quite different between animals. Interestingly, all four animals damaged in the early-season cohort had a shorter axial length than any animal in the remaining cohorts (although the one animal with damage observed in the late-season cohort (DM_204304) had the second *longest* axial length across animals). Future experiments may need to explore potential seasonal variations in axial length among the 13-LGS or include using axial length as an additional control when assigning seasonal cohorts in longitudinal studies.

Multiple notable limitations to our study exist. These include inherent variability associated with external factors while injecting intravitreal phototoxic drugs influencing the volume of reflux after intravitreal injection between animals or chemical exposure to the retina. Animal eye position in relation to gravity was limited to the brief period postinjection (∼1 minute), where the animal eye remained vertical relative to gravity (also to control for the potential backflow). Animals remained in generally the same position until they recovered from anesthesia. Other studies have utilized techniques, such as self-sealing hyaluronic acid–coated needles, to limit the amount of vitreous and chemical reflux postinjection.^37^ Other alternative approaches to chemical delivery, such as preretinal injections, have been used in the 13-LGS and may produce more consistent retinal degeneration, regardless of the time of season.^38^ Alternatively, deep vitreous injections guided by intraoperative OCT may account for potential variability in administration methods between animals.^39^ Additionally, the small sample sizes in our study make adequate comparisons between cohorts challenging. Furthermore, monitoring normal physiological changes, including test bouts of torpor in mid- or late season, which occur in the 13-LGS throughout euthermia, would be beneficial.^40^

Our results have implications for future studies considering using chemical models for retinal degeneration in the 13-LGS, particularly intravitreal methods. As seen in other studies, the use of ATP as a chemical model in the 13-LGS is supported by the absence of systemic complications after intravitreal injection.^7^^–^^9^^,^^25^ However, variability in the response to ATP seen here suggests that intravitreal ATP at the dosages used may not be a reliable model of producing consistent, selective photoreceptor degeneration in the 13-LGS. Although intravitreal injection as a delivery method may be more practical within the 13-LGS, alternative means of administration should be considered. The creation of a robust, reliable animal model of selective photoreceptor degeneration remains important for evaluating the efficacy of neurodegenerative therapies.

## Supplementary Material

Supplement 1

Supplement 2

Supplement 3

Supplement 4
