# Supplementary material for: Seasonal Variation in ATP-Induced Retinal Damage in the Cone-Dominant 13-Lined Ground Squirrel
Source: Transl Vis Sci Technol. 2024 Nov 7;13(11):5. doi: 10.1167/tvst.13.11.5 (PMC11547255; doi:10.1167/tvst.13.11.5)
Supplement: Supplement 1 [file tvst-13-11-5_s001.pdf]

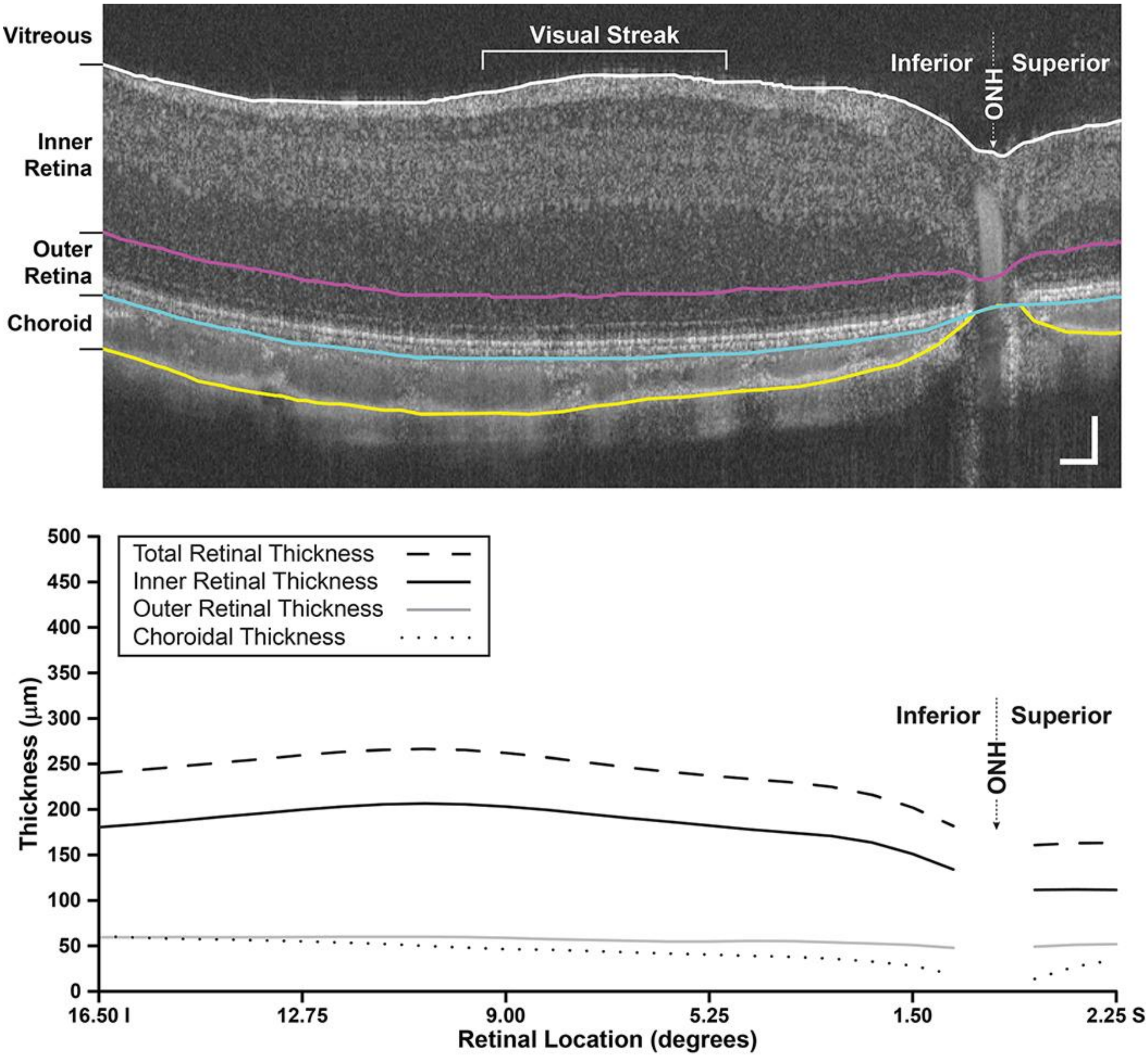

Supplementary Figure S1. Example of OCT retinal lamination boundaries. Shown are generated thickness profiles for the 13-LGS retina. **TOP.)** OCT line scan of a control animal with an example of retinal lamination barriers, generated from DOCTRAP, used to estimate retinal layer thickness from 2° superior retina to 16.50° inferior retina within each scan, including: Total retina, Inner retina, Outer retina and Choroidal thickness. Scale bar is 1 deg (lateral) and 50 µm (axial). **BOTTOM.)** Graph representing average thickness for each of the four layers analyzed from 2.25° superior retina to 16.50° inferior retina (excluding the optic nerve head, ONH).
