# Supplementary material for: Seasonal Variation in ATP-Induced Retinal Damage in the Cone-Dominant 13-Lined Ground Squirrel
Source: Transl Vis Sci Technol. 2024 Nov 7;13(11):5. doi: 10.1167/tvst.13.11.5 (PMC11547255; doi:10.1167/tvst.13.11.5)
Supplement: Supplement 2 [file tvst-13-11-5_s002.pdf]

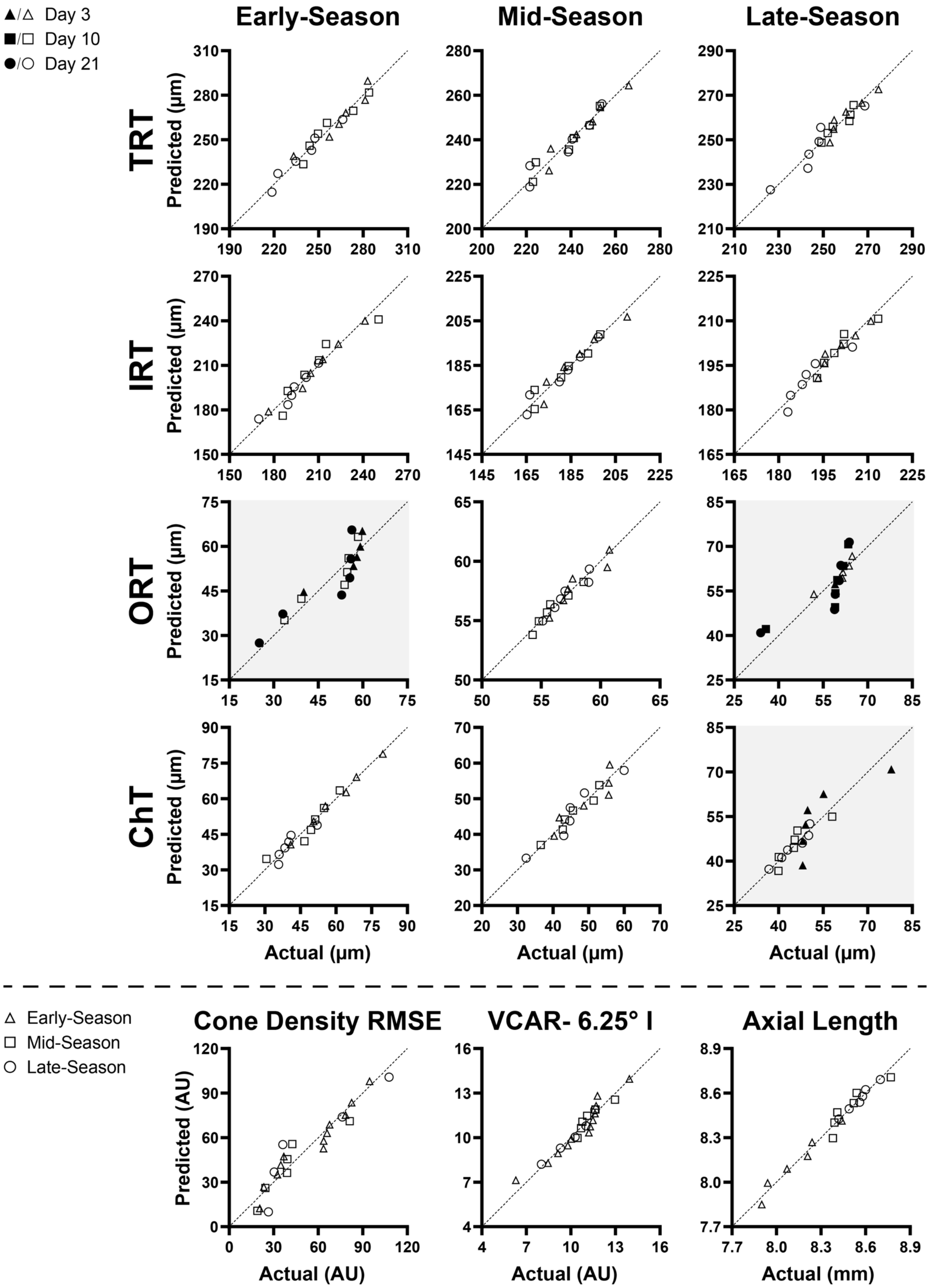

Supplementary Figure S2: Retinal and Choroidal Thickness, Cone Density RMSE, VCAR, and Axial Length Q-Q Plots. Visualization of data distribution on Q-Q plots supports Shapiro-Wilk assessments of non-normal distributions for three datasets. Filled symbols on the shaded plots indicate data that failed Shapiro-Wilk normality testing.
