# Supplementary material for: Seasonal Variation in ATP-Induced Retinal Damage in the Cone-Dominant 13-Lined Ground Squirrel
Source: Transl Vis Sci Technol. 2024 Nov 7;13(11):5. doi: 10.1167/tvst.13.11.5 (PMC11547255; doi:10.1167/tvst.13.11.5)
Supplement: Supplement 3 [file tvst-13-11-5_s003.pdf]

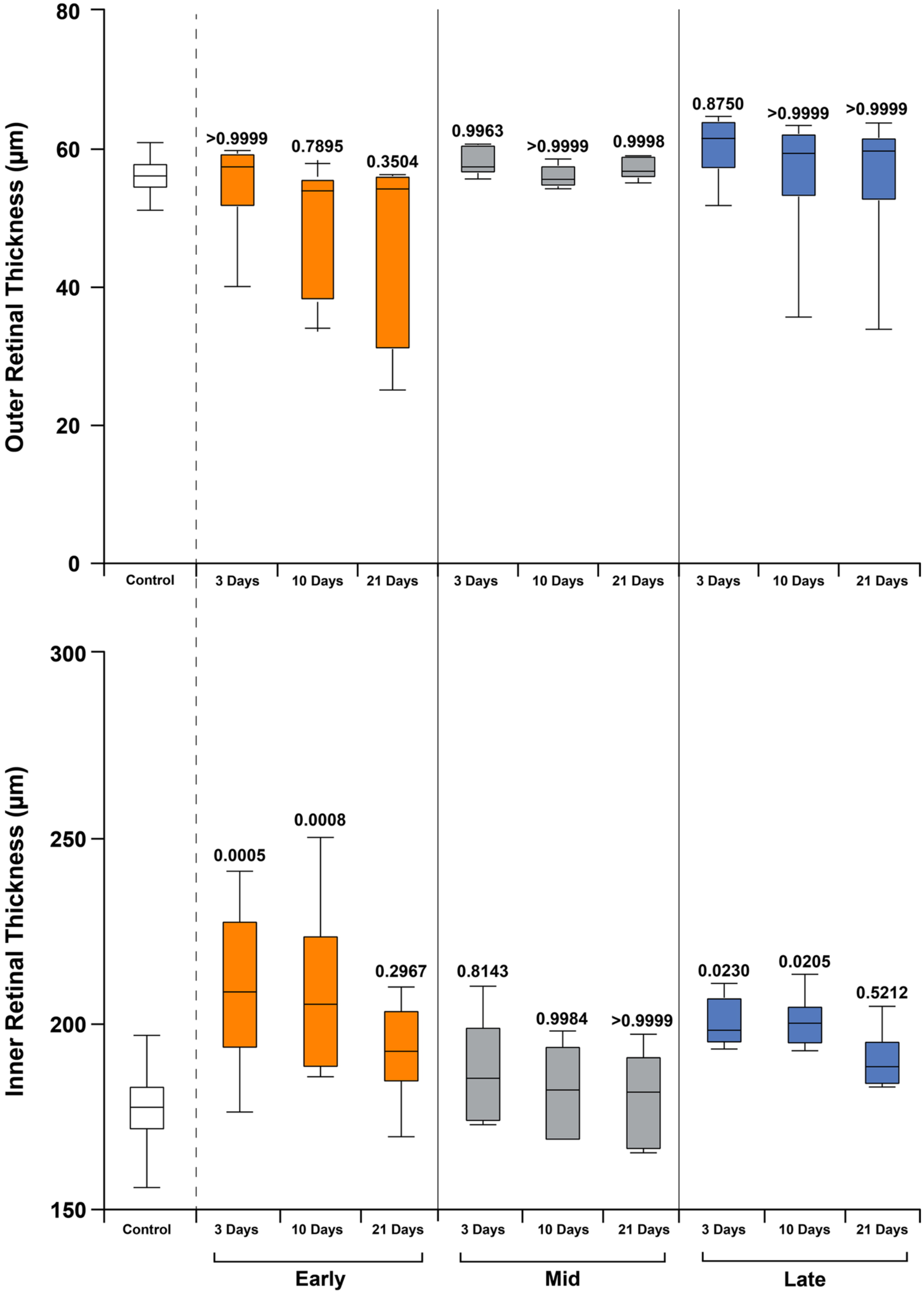

Supplementary Figure S3. Changes observed in TRT due to increases in inner retinal thickness Changes in total retinal thickness appear due mainly to changes in the inner retinal layers. No significant differences between average outer retinal thickness in early-, mid-, and late-season groups throughout the follow-up period, 3-, 10-, and 21-days, were observed. Both early- and late-season animal average inner retinal thickness were significantly different than controls at 3-, and 10-days, while values at 21-day follow-up were not. Differences in mid-season average inner retinal thickness were not significantly different from controls throughout follow-up. Individual p values are shown on the graph, generated using two-way ANOVA.
