# Supplementary material for: Seasonal Variation in ATP-Induced Retinal Damage in the Cone-Dominant 13-Lined Ground Squirrel
Source: Transl Vis Sci Technol. 2024 Nov 7;13(11):5. doi: 10.1167/tvst.13.11.5 (PMC11547255; doi:10.1167/tvst.13.11.5)
Supplement: Supplement 4 [file tvst-13-11-5_s004.pdf]

			Early			Mid			Late		
**TRT**	**Control Average RMSE**	11.5203	**Early**			**Mid**			**Late**		
	**95%CI (upper bound)**	22.0158	**3 Days**	**10 Days**	**21 Days**	**3 Days**	**10 Days**	**21 Days**	**3 Days**	**10 Days**	**21 Days**
	**Animal #1**		11.5145	11.0091	13.3696	4.9480	10.7488	12.9269	27.9665	19.6978	16.0782
	**Animal #2**		37.6001	22.4802	6.2581	12.2338	10.4954	8.7854	42.4322	31.9443	10.6654
	**Animal #3**		50.3482	55.6275	35.7953	21.6965	22.8502	15.7478	22.4738	21.4463	17.4495
	**Animal #4**		50.5662	42.4818	34.0021	13.7075	15.9254	16.4380	22.7170	25.2969	15.7311
	**Animal #5**		31.1046	22.9177	14.7370	35.2043	22.5263	22.9851	23.1707	29.1946	11.2788
	**Animal #6**		24.7738	13.2465	15.9933	17.4366	9.5096	8.5628	34.6309	29.4477	36.8253
**ChT**	**Control Average RMSE**	7.2479	**Early**			**Mid**			**Late**		
	**95%CI (upper bound)**	15.73	**3 Days**	**10 Days**	**21 Days**	**3 Days**	**10 Days**	**21 Days**	**3 Days**	**10 Days**	**21 Days**
	**Animal #1**		13.1138	4.2402	7.2900	6.2839	9.4504	14.1677	7.1159	16.3589	5.5512
	**Animal #2**		22.8323	6.7465	5.5487	13.5896	4.6323	6.4340	7.6344	5.1808	3.6018
	**Animal #3**		38.8153	12.2958	9.0061	12.5385	10.9708	17.8252	7.0646	4.1746	7.5632
	**Animal #4**		30.2317	21.8405	12.6237	2.7896	3.9325	2.3008	36.9846	4.7826	8.0673
	**Animal #5**		8.6842	8.9283	3.3551	13.7723	9.3498	2.5710	5.9742	3.9732	2.4676
	**Animal #6**		4.0978	14.4558	8.6443	6.0719	3.6057	3.6758	12.2243	3.7624	8.2881
**IRT**	**Control Average RMSE**	10.3553	**Early**			**Mid**			**Late**		
	**95%CI (upper bound)**	19.73	**3 Days**	**10 Days**	**21 Days**	**3 Days**	**10 Days**	**21 Days**	**3 Days**	**10 Days**	**21 Days**
	**Animal #1**		10.4957	12.2124	10.4230	4.8938	10.0368	13.1433	19.4365	16.9471	11.3729
	**Animal #2**		36.4454	34.9959	26.8466	10.0877	7.6788	6.8790	34.5451	25.8789	7.9461
	**Animal #3**		65.7469	75.1812	29.4592	20.4602	21.8819	12.8206	20.1943	19.2926	13.8458
	**Animal #4**		46.7929	40.2782	33.9641	13.1627	14.5353	14.2639	26.3931	41.3381	19.3055
	**Animal #5**		27.9570	23.7449	15.4701	35.8533	23.7739	23.0036	17.4069	25.6269	7.9773
	**Animal #6**		22.8088	13.4888	15.3620	13.4757	7.9574	7.5891	29.6343	22.3293	29.0223
**ORT**	**Control Average RMSE**	3.06285	**Early**			**Mid**			**Late**		
	**95%CI (upper bound)**	5.42	**3 Days**	**10 Days**	**21 Days**	**3 Days**	**10 Days**	**21 Days**	**3 Days**	**10 Days**	**21 Days**
	**Animal #1**		2.4835	2.7565	3.8135	1.4985	2.5822	1.3496	9.4085	4.1196	5.1655
	**Animal #2**		8.4288	19.9425	27.5582	4.9682	3.8009	4.2978	8.5256	6.2355	4.4153
	**Animal #3**		19.4906	25.2465	32.8232	2.2101	2.7701	3.6960	3.7417	4.2799	3.9686
	**Animal #4**		4.2985	3.2235	2.8456	2.1444	2.8605	2.9533	7.3350	23.5295	25.2158
	**Animal #5**		3.4309	2.1054	1.9364	3.6168	2.6807	2.6837	6.1398	4.1194	4.6688
	**Animal #6**		2.3820	2.9502	1.9151	5.0558	2.5155	1.4902	5.7005	8.1448	8.2196

Supplementary Table S1. Calculated RMSE values by animals within each cohort. Calculated average RMSE and 95% confidence interval (CI) above the mean (mean + 1.96*SD) for total retinal thickness (TRT), choroidal thickness (ChT), inner retinal thickness (IRT), and outer retinal thickness (ORT), compared to each animal within early-, mid-, and late-season cohorts across follow-up post-injection at 3-, 10-, and 21-days.
